# PReS-FINAL-2042: Health-related quality of-life in Turkish children and adolescents with juvenile idiopathic arthritis

**DOI:** 10.1186/1546-0096-11-S2-P55

**Published:** 2013-12-05

**Authors:** E Tarakci, SN Baydogan, O Kasapcopur

**Affiliations:** 1 Division of Physiptherapy and Rehabilitation, Faculty of Health Science, Istanbul University, Turkey; 2 Department of Pediatric Rheumatology, Medical Faculty of Cerrahpasa, Istanbul University, Istanbul, Turkey

## Introduction

Juvenile idiopathic arthritis (JIA) in children and adolescents is a chronic autoinflammatory affection which might ocur in any joint. The activity and participation restrictions that result from the arthritis and extra-articular manifestations can negatively impact the child's quality of life. Therefore, it is important to assess the quality of life of Turkish children and adolescents with JIA. Furthermore, health-related quality of-life measurement has been increasingly integrated into clinical trials, clinical practice improvement initiatives, and health care services research and evaluation as an essential health outcome.

## Objectives

The aim of this study was to assess quality of life in Turkish children and adolescents with JIA.

## Methods

A total of 212 (female = 149, male = 63) patients with JIA and their parents were enrolled in the study. The subjects were recruited in a pediatric rheumatology clinic. They were diagnosed with JIA by a pediatric rheumatologist based on the clinical criteria of the International League of Associations for Rheumatology (ILAR). The inclusion criteria included children between the age of 2 and 18 years with JIA. Patients with recent diagnoses of JIA and those with psychiatric and mental deficits were excluded. The Turkish version of PedsQL 3.0 Arthritis Module was used to evaluate quality of life.

## Results

The mean age was 9.02 ± 4.38 years (range 2-18 years). The mean disease duration was 3.92 ± 3.26 years (range 0.5-15 years). Patient population consisted of 106 (51.2%) patients with polyarticular arthritis, 89 (41.1%) patients with oligoarticular arthritis, 11 (4.9%) patients systemic arthritis and 6 (2.9%) patients psoriatic arthritis subtype. Total score of PedsQL-self report was 78.92 ± 12.52 for 5-7 years, 75.14 ± 16.45 for 8-12 years, 77.37 ± 13.08 for 13-18 years. Total score of PedsQL-parent's report was 70.73 ± 17.05 for 2-4 years, 72.43 ± 12.96 for 5-7 years, 68.81 ± 17.14 for 8-12 years and 75.19 ± 16.84 for 13-18 years. There was no statistically significant difference for PedsQL parent's report total scores in JIA subtypes (p > 0.05). There was statistically significant difference for PedsQL parent's report (daily activities scores, treatment scores and communication scores) between age groups (p < 0.05).

## Conclusion

This study showed that the results of the quality of life in Turkish children with JIA. The quality of life has decreased when the age increases in present study. The decrease in quality of life are not correlate with subtype of JIA. However, it may relate to sequelaes in later stages of the disease, illness perceptions, and reduced expectation of treatment in JIA.

## Disclosure of interest

None declared.

